# 3D Oxidized Graphene Frameworks for Efficient Nano Sieving

**DOI:** 10.1038/srep21150

**Published:** 2016-02-19

**Authors:** Pranav Bhagwan Pawar, Sumit Saxena, Dhanashree Kamlesh Badhe, Raghvendra Pratap Chaudhary, Shobha Shukla

**Affiliations:** 1 Nanostructures Engineering and Modeling Laboratory, Department of Metallurgical Engineering and Materials Science, Indian Institute of Technology Bombay, Mumbai, 400076, India

## Abstract

The small size of Na^+^ and Cl^−^ ions provides a bottleneck in desalination and is a challenge in providing alternatives for continuously depleting fresh water resources. Graphene by virtue of its structural properties has the potential to address this issue. Studies have indicated that use of monolayer graphene can be used to filter micro volumes of saline solution. Unfortunately it is extremely difficult, resource intensive and almost impractical with current technology to fabricate operational devices using mono-layered graphene. Nevertheless, graphene based devices still hold the key to solve this problem due to its nano-sieving ability. Here we report synthesis of oxidized graphene frameworks and demonstrate a functional device to desalinate and purify seawater from contaminants including Na^+^ and Cl^−^ ions, dyes and other microbial pollutants. Micro-channels in these frameworks help in immobilizing larger suspended solids including bacteria, while nano-sieving through graphene enables the removal of dissolved ions (e.g. Cl^−^). Nano-sieving incorporated with larger frameworks has been used in filtering Na^+^ and Cl^−^ ions in functional devices.

Extraordinary properties of graphene make it a potential candidate for nano-sieving in desalination. Molecular dynamics simulations suggests better salt rejection in graphene oxide based frameworks than existing technologies^1^. Synthesis of graphene based materials using conventional methods is expensive, has large energy footprints and resource intensive. We have synthesized 3D oxidized graphene frameworks by facile green method, fabricated their membranes and demonstrate filtration of small ions including Na^+^ and Cl^−^ from sea water along with its purification. Optical, chemical and electrical measurements reveal that these frameworks filter the solution efficiently by utilizing sieving at micro and nano scale. Filtration capability is significantly better than that of mono-layer graphene membranes. Membranes are easy to fabricate, reusable and economically viable. This is expected to have wide implications in meeting the demand for fresh water, a matter of global concern, at lower cost^2^ by desalination and water purification especially for point of use application.

The small size of Na^+^ and Cl^−^ ions has created a bottleneck in the development of filtration devices to filter these ions from saline solution. De-ionization of saline water is achieved by thermal^3^ and membrane based processes^4^. Both of these categories involve processes that are resource intensive, capital intensive^5^ and have large energy footprints. Recently, membranes using different materials such as activated carbon fibers^6^, carbon nanotubes^7^, graphene^8^,^9^,^10^ and graphene oxide^11^ have been explored. All these carbon derivatives provide large, active surface area for electrosorption or chemisorption of ions and pollutants^12^. Nanoporous graphene with hydrogenated pores have been suggested as a better replacement for reverse osmosis membrane for desalination^8^,^11^,^12^,^13^,^14^. They provide large surface area and active sites for immobilization and thinness for better flux. However practical realization of mono-layered graphene sheet is impeded by leakage^10^ and are mostly ineffective in filtering Na^+^ ions. As an alternative to expensive graphene based filters, green methods are being used to synthesize graphene based materials^15^. Attempts are also being made to use plant xylem containing conduit linked via submicron pits to filter water^16^. However, these xylem filters prepared from freshly cut sapwood were found to be effective only for filtering larger dye molecules and e-coli^16^. Unfortunately none of these options provide efficient and economically feasible solution for filtering Na^+^ and Cl^−^ ions for the production of fresh water from saline source at industrial or individual level; hence there is an urgent need to address this global issue^12^,^17^.

## Results and Discussion

**Figure 1:**
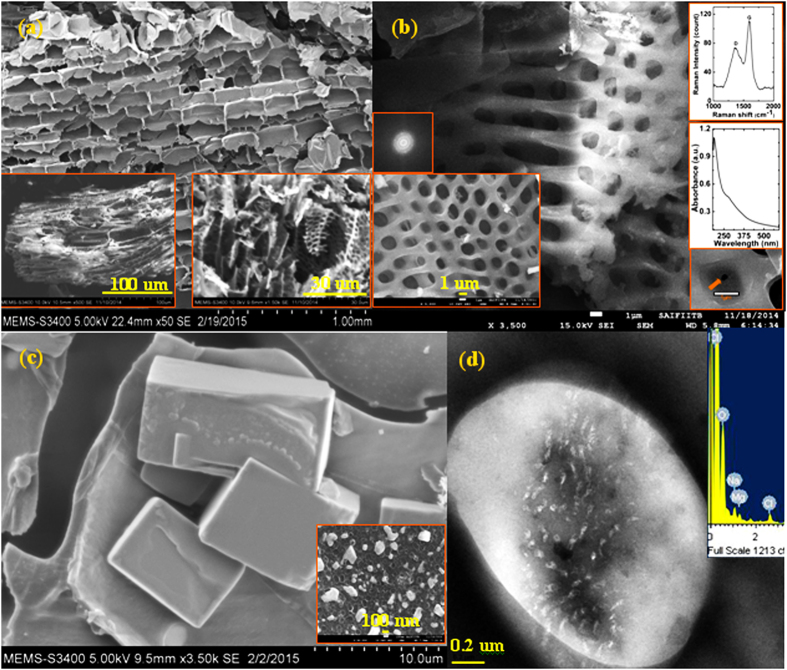
Micro structural analysis of porous membranes fabricated from 3D oxidized graphene framework (**a**) Cross-sectional SEM micrograph of graphene based filter showing micro-pores. Bottom-left inset shows micrograph of large chunk while the bottom-right inset shows a zoomed in image of a part of the same (**b**) SEM micrograph shows 3D view of a lower dimensional microhole array inside larger microhole structure. Inset on the left shows the front surface of similar such structure while the inset on the right shows the Raman spectrum (top-right), UV-Vis absorption spectrum (middle-right), zoomed in image of nanohole within microhole (bottom-right) and the FFT image of sheet (top-left). (**c**) Shows large (~10 μm) NaCl crystallites sticking on the membrane after filtration. The inset at the bottom-left shows smaller (<200 nm) NaCl crystals on the surface of membrane. (**d**) TEM micrograph of clusters of NaCl nano crystals on membrane. The EDX confirms the presence of Na and Cl elements.

The 3D oxidized graphene framework used in this study was synthesized by charring fresh sugarcane bagasse for about 10 minutes. Thermal processing in air leads to slight oxidation of the 3D graphene frameworks in Fig. 1. As these are not fully oxidized graphene sheets, hence not referred to as graphene oxide frameworks in this manuscript. The oxidized graphene sheets were characterized by the presence of ‘D’ and ‘G band at with I_D_/I_G_ ratio of ~0.6471 in their Raman signature shown in top right inset in Fig. 1(b). Systematic studies of such defects using Raman spectroscopy^18^ have shown that I_D_/I_G_ ratio can be used to study point like defects in graphene, thereby suggesting the presence of point like defects in the oxidized graphene membrane which act as nanopores for sieving of small ions. This is also supported by UV-Vis signature at ~270 nm^19^ of partially oxidized graphene sheets in middle inset on the right which is red shifted from the peak of graphene oxide peak at ~233 nm^20^ suggesting that the conjugation of π electron is mostly restored in these 3D networks as compared to graphene oxide where it is mostly disrupted. The material comprising of 3D oxidized graphene frameworks was compressed in a die to fabricate membranes. Micro-structural imaging using SEM was performed by crushing the membrane as shown in Fig. 1 to understand the filtration process.

Analysis of the micrograph suggest that the thermal processing of sugarcane bagasse produces interconnected hierarchical porous structures forming a 3D network of micro-channels made of oxidized graphene. Formation of diffused hexagonal pattern in FFT micrograph is seen in bottom right inset in Fig. 1(b). This modifies the fluid transport system inherited from bagasse leading to formation of highly torturous path which assist in filtering of solutes from the saline solution. It needs to be emphasized that any ordered arrangement of micro- or meso-porous structures as present in xylem will provide access to freeways for flow of water resulting in easy escape for solute particularly small ions and molecules. The randomly aligned hierarchical structure of sieves in Fig. 1 enables in the filtration of the feed solution both at micro as well as nano scales. The porous 3D framework in these linked structures not only plays an important role of immobilizing ions^21^, bacteria and living organisms; but also enable in trapping of large salt crystals by providing torturous path to the flow of feed water^21^. These also provide larger interaction of material surface with saline/contaminated feed water containing dissolved ions and other solutes. Hydroxyl groups in sucrose which forms a major component of sugarcane juice present in the sugarcane bagasse are hydrophilic in nature. Some of these groups survive and get attached in the vicinity of point like defects in graphene sheets obtained from sugarcane juice^22^ during processing. The presence of these functional groups assists the flow of water molecules through the point like defects forming nanopores in intrinsically defected graphene sheets. These nanopores thus acts as sieves for blocking the passage of small molecules and ions as suggested by molecular dynamics studies^11^. Filtration of salt nanocrystals through slit pores were also observed in some of the TEM micrographs. Thus the membranes so formed from 3D oxidized graphene frameworks utilize the virtues of both micro and nano sieving processes allowing a multi-scaled sieving process.

Microscopic analysis of the membranes after filtration of 0.5 M NaCl solution in Fig. 1(c) shows the presence of larger salt crystals entangled on the surface of the oxidized graphene sheets. Since the NaCl solution does not contain any suspended particles, the origin of these nano-micro NaCl crystals is understood to be due to growth from random nucleation sites that are produced during the filtration process. Figure 1(d) shows small NaCl grains (<50 nm) which were identified by Na and Cl peaks in the EDX spectrum shown in the inset. The filtration of halides is confirmed by disappearance of the absorption peak corresponding to halides in the comparative UV-Vis absorption spectrum of the filtrate and feed sea water (supplementary figure S2).

Standard parameters quantifying water quality are conductivity, TDS, and salinity. The filtrate obtained using fabricated porous graphene filters were tested for these parameters using 0.5 M NaCl solution in milli-Q water as well as seawater for filtration of Na^+^ and Cl^−^ ions at room temperature under atmospheric pressure. Results suggest that the filtrate obtained using 3D oxidized graphene membranes qualify to be labeled as fresh water. The filtrate obtained using sea water as feed was also tested for different elemental solvents such as chlorides, residual chlorine, phosphorous and fluorides (supplementary information Table T1). These tests show that the 3D oxidized graphene framework membranes are extremely effective in these elemental solvents to levels much better than the acceptable limits of fresh water.

**Figure 2:**
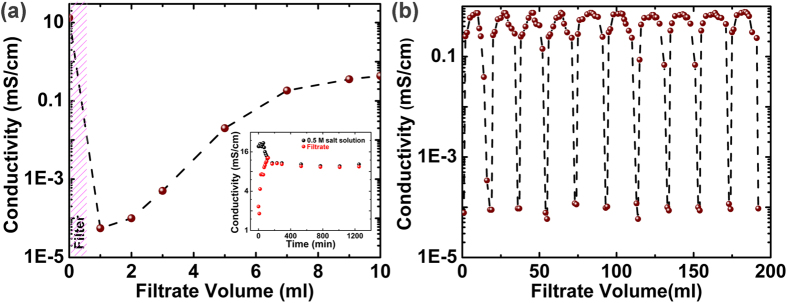
(**a**) Conductivity test of filtrate obtained by filtering 0.5 M NaCl solution. The inset shows the permeability of the filter, plotted on logarithmic scale. (**b**) Recycling performance of the filter shows that the filter’s salt rejection performance remains almost unaltered even after ten cycles of filtration.

Conductivity measurements were performed as shown in Fig. 2(a) to quantify the efficiency of the membranes. It is observed that the conductivity of filtrate initially reduces by approximately six orders of magnitude during filtration and subsequently increases as the filtrate volume increases. This can be understood by permeation studies, which suggest that once the active sites in intrinsically defective graphene sheet in the filter get saturated the conductivity starts to increase with further flow of solution more and more nanopores gets blocked and the conductivity becomes almost constant. The conductivity approaches to approximately two orders of magnitude less than that of feed water and lies comfortably within the admissible range of typical drinking water. The filter was recycled 10 times by rinsing it with fresh water as shown in Fig. 2(b) and conductivity measurements were performed. It is observed that the conductivity of the filtrate remains almost constant even after recycling the membrane ten times. The amount of salt removed from the source was estimated from the conductivity curve by using the equation 1.


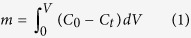


It is observed that ~29.20 mg/ml (99.95%) of NaCl is filtered in the 1^st^ ml of the filtrate which decreases to ~25.59 mg/ml (97.34%) at the end of filtering 10 ml and approaches to ~13 g (91.02%) after filtering 490 ml of 0.5 M NaCl solution (supplementary figure S3). The NaCl rejection capacity of these membranes are approximately 66.3 mg/g (after filtering 490 ml of water) and is significantly higher than that achieved using activated carbon (2–20 mg/g) and capacitive deionization (5 mg/g)^7^. Permeation studies were performed by observing the change in conductivity of the liquid in the columns filled with NaCl solution and milli-Q water respectively. The flow rate of ~0.013 Lm^−2^h^−1^ was observed for 0.5 M NaCl solution corresponding to an osmotic pressure of ~22.5 bar at room temperature. A device was fabricated (supplementary figure S1) using synthesized 3D oxidized graphene framework membrane to assess the feasibility of using these devices for point of use applications. It is observed that these devices are capable of desalinating sea water with total dissolved solid concentration (TDS) >30,000 ppm to <990 ppm which lies in the category of fresh water. These were also found to be efficient in filtering dye molecules completely (supplementary figure S4).

**Figure 3:**
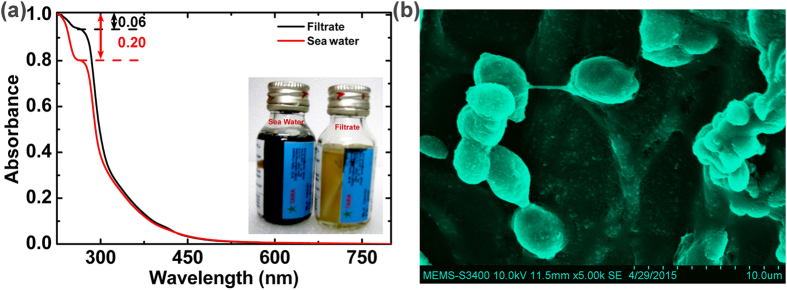
(**a**) UV-Vis spectrum of the water samples bottle for fecal pollution test for sea water and filtrate obtained from porous graphene filters. The inset shows visible color change in “Aquacheck” water testing kit (**b**) SEM micrograph of the porous graphene filter surface showing immobilized e-coli bacteria on the surface of the porous graphene filter.

Filtrate obtained from membranes was tested using commercial water testing kits for filtering bacteria and other micro-organisms linking to water borne diseases due to fecal pollution. Visual test (Fig. 3a) shows that these membranes are capable of filtering bacteria and other microorganisms from contaminated water samples. Samples from commercial water filters (RO + UV) and tap water tested using this kit (supplementary information figure S5) were found to be of equivalent quality as the filtrate obtained from the processed porous graphene filters. The absorption edge at 230 nm corresponds to the DNA of the live bacteria^23^. It is observed that the ratio of absorbance intensity I_260_/I_230_ (Fig. 3a) reduces significantly for filtrate from processed filters. This suggests the almost all the bacterium/ micro-organism gets filtered out during the filtration process.

## Conclusion

To conclude, we have synthesized 3D oxidized graphene framework using green method. The membrane fabricated using these frameworks are highly efficient in removing Na^+^ and Cl^−^ ions along with other contaminants such as dye molecules and fecal pollutants including e-coli bacteria. The 3D oxidized graphene frameworks arranged in random closely packed manner provides a torturous path to assist in filtration. These membranes utilize the virtues of both micro and nano sieving processes. The NaCl rejection capacity of these membranes was greater than 90% even after filtering more than 500 ml of 0.5 M salt solution. The membranes are extremely effective in removing chlorides, residual chlorine, phosphorous and fluorides from sea water (TDS > 30000) as feed solution to the levels much better than the acceptable limits of fresh water (TDS < 990). These are not only simple to synthesize and easy to fabricate and recyclable but also highly economical to use in point of use applications.

## Methods

Porous 3D oxidized graphene membranes (~1.2 cm diameter) were prepared by thermally processing for device fabrication. The pellet was sandwiched between two plastic filters with ~1 mm^2^ holes to provide structural stability from flow of tap water. This assembly was finally mounted inside a plastic case. Microstructural characterizations were performed using TEM and FEGSEM. Raman spectroscopy was performed using Witec alpha 300RAS confocal-raman imaging system using 532 nm laser for excitation. The UV-Vis spectrum was obtained using shimadzu UV2600 UV-Vis spectrophotometer. Water quality tests were performed using commercial test kits “Aquacheck”. The details vital parameters test for evaluating the health of water has been provided in supplementary information. Conductivity measurements and permeability studies were performed using standard laboratory equipments.

## Additional Information

**How to cite this article**: Pawar, P. B. *et al.* 3D Oxidized Graphene Frameworks for Efficient Nano Sieving. *Sci. Rep.* **6**, 21150; doi: 10.1038/srep21150 (2016).

## Supplementary Material

Supplementary Information
