# Supplementary material for: 3D Oxidized Graphene Frameworks for Efficient Nano Sieving
Source: Sci Rep. 2016 Feb 19;6:21150. doi: 10.1038/srep21150 (PMC4759691; doi:10.1038/srep21150)
Supplement: Supplementary Information [file srep21150-s1.doc]

**Supplementary Information**

Pranav Bhagwan Pawar, Sumit saxena, Dhanashree Kamlesh Badhe, Raghvendra Pratap Chaudhary and Shobha Shukla^*^

*Nanostructures Engineering and Modeling Laboratory, Department of Metallurgical Engineering and Materials Science, Indian Institute of Technology Bombay, Mumbai, India, 400076*

* [sshukla@iitb.ac.in](mailto:sshukla@iitb.ac.in)

**Sample details:-**

The seawater used for sampling was collected from the Arabian Sea at Girgaon Chowpatty sea beach in Mumbai, India.

0.5M solution was prepared by mixing 29.22139 g of NaCl in 1 liter of milli-Q water.

**Experimental setup for desalination:-**

The filter was fitted in a small vertical column and a collection conical flask was placed below the column. Before filtering, the membrane was rinsed with normal tap water to remove caramelized sugarcane juice. The salt/sea water was filtered at room temperature and normal pressure.


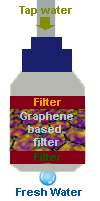

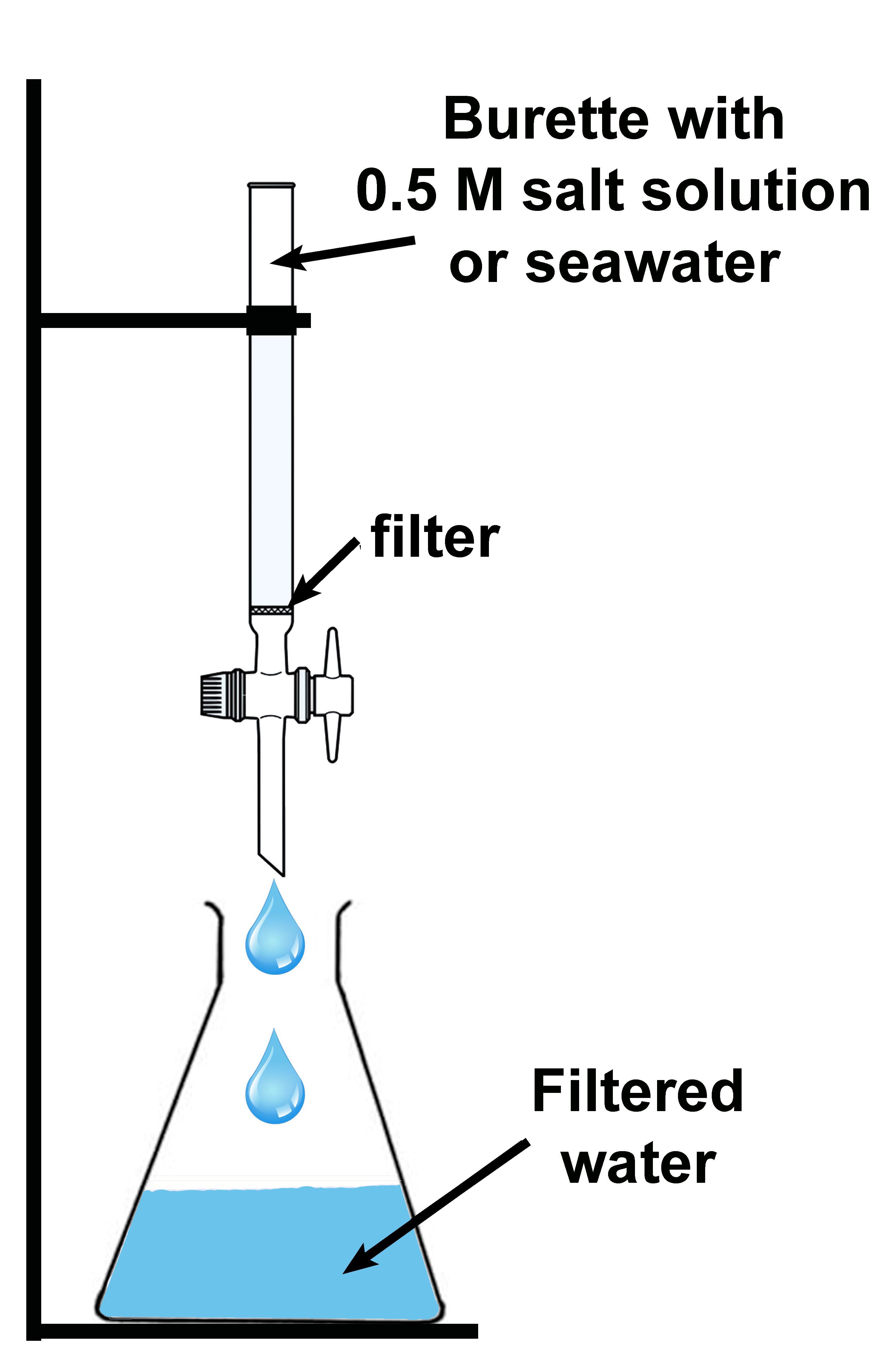

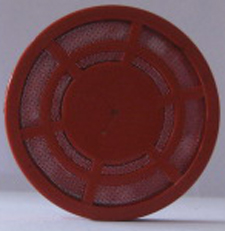


**Figure S1:-** Filtration setup used for filtering salt/sea water at room temperature and under normal pressure. Inset shows the cross-section of a working device attached to tap with normal flow of water. The inset at the bottom shows the top view of the plastic filter used at the top and bottom of the porous graphene filter to provide structural stability to the porous graphene filter to withstand the pressure of water flow in the tap.

**UV-Vis absorption of Feed sea water and Filtrate**

**Figure S2:-** UV-Vis absorption spectrum of the sample water before and after filtering. The peak ~250nm corresponds to NaCl. The filtered sample shows almost no peaks corresponding to NaCl and HOCl.

**Standard water parameters of the filtered sample using water testing kit:-**

The following important parameters were tested using standard water testing kit provided by CTARA, IIT Bombay to test the purity of filtered water.

**Table T1:-** Standard water test parameters of drinking water and their permissible limits.

| Parameters | Sea water | Filtrate | Permissible limits |
| --- | --- | --- | --- |
| Temperature | 26 C | 26 C |  |
| Residual Chlorine | ~0.5mg/l | ~0mg/l | ~0.2mg/l |
| Phosphorus | 0.5mg/l | 0.1mg/l | ~5mg/l |
| Hardness | 7200mg/l | 320mg/l | 300 – 600mg/l |
| Chlorides | 35451mg/l | 35.41mg/l | 250-1000mg/l |
| Fluorides | 1.5mg/l | 0.6mg/l | ~1-1.5mg/l |
| Ammonia | 2 mg/l | 1mg/l | ~1.5mg/l |
| Nitrate | ~0 mg/l | ~10mg/l | ~45mg/l |

The details of the methodology of the tests are given below:-

1. **Residual Chlorine**:- HOCl and OCl appears transiently in surface or ground water. O-Toludine forms yellow complex, the intensity of which depends on amount of residual chlorine in the sample. The data was compared to standard data available.
2. **Chloride in form of Cl ions**:- Chlorine ions will form a precipitate of silver chloride in a natural or slightly alkaline solution upon titration with standard silver nitrate. Potassium chromate is used as an indicator. 5ml of sample was taken and 1 drop of potassium chromate was added and the resulting solution was titrated with silver nitrate to obtain brick red color. The amount of chloride will be volume of silver nitrate consumed x 354.5 mg/l.
3. **Nitrates as ions:-** Nitrates react with phenoldisulphonic acid (PDA) and produces a nitro-derivative which, in an alkaline solution develops yellow color with its intensity being dependent on concentration. 5 ml of sample was taken, dried and cooled to room temperature. 8-9 drops of PDA was added and stirred. Solution was diluted by adding 2ml of milli-Q water was added. Subsequently 23 drops of Ammonia solution was added. The solution was further diluted to make the total volume to 5ml and results were compared to standard data.
4. **Fluoride:-** Fluorides present in sample react with colored zirconium dyes and bleaches the color of the dye forming ZrF6, the intensity of the emission of dye decreases with increasing fluoride concentration. 50 ml of sample solution was taken and 2.5 ml of Zirconyl Alizarine was mixed and aged for one hour and was compared to standard data.
5. **Phosphorous:-** Phosphorous reacts with ammonium molybdate forming molybdophosphoric acid. This is reduced by stannous chloride to form intensely colored molybdenum blue. 12 ml of sample was taken and mixed with 0.4ml of Ammonium molybdate. 2 drops of stannous chloride was further mixed to this and reaction allowed for 10 minutes. The data was compared to the standard data.
6. **Hardness:-** is the property of water which prevents the lather formation when using soap and increases the boiling point of water and principally due to calcium and magnesium cations. This depends on the ability of the EDTA or its disodium salt to form stable complexes with calcium or magnesium ions. When the dye eriochrome black-T is added to a solution containing calcium, magnesium ions (pH10) form a red wine complex. This solution is titrated with a standard solution of EDTA, which extracts Calcium and magnesium from dye complex and the dye is changed back to original blue color. 5ml of sample was taken and 1-2 drops of Ammonia buffer was added to raise the pH to 10. A pinch of Eriochrome black-T powder was added. This solution was titrated with EDTA solution till color changes from wine red to blue.
7. **Ammonia:**- Nesslers reagent when added to ammonia forms orange-brown product in colloidal form but flocculates on standing for prolonged duration. 5ml of sample was taken and 2-3 drops of Nessler’s reagent was added and data compared to standard data.

**Feasibility studies of processed filter for point of use applications:-**

The filtration capacity of a single filter was determined by checking the conductivity of the solution after filtering every 20 ml of 0.5M salt solution. The conductivity of the filtered solution almost becomes constant. This suggests that the salt rejection efficiency remains almost constant after filtering first 10 ml of salt solution.

**Figure S3:-** Plot of conductivity of filtrate with increasing volume. The experimental data has been joined to act as guide for the eye.

**Removal of dye molecules using Oxidized graphene frameworks**

**Figure S4:-** % dye removal with increasing volume of solution. The experimental data has been joined to act as guide for the eye

**Test for Faecal Pollution**:-

The presence of coliforms in drinking water associated with organisms produce hydrogen suphide. Enteric bacteria such as salmonella, Proteus, Citrobacter and some strains of Klebsiella also produce H_2_S. 20 ml of sample (sea water and filtrate) was taken in two different “Aquacheck” bottle (provided by CTARA, IIT Bombay as a part of water testing kit) and the lids were closed immediately after transferring the samples. The bottles were allowed to stand at room temperature for more than 48 hrs. The sample water from the sea turns deep black in color, while the filtrate becomes yellowish.


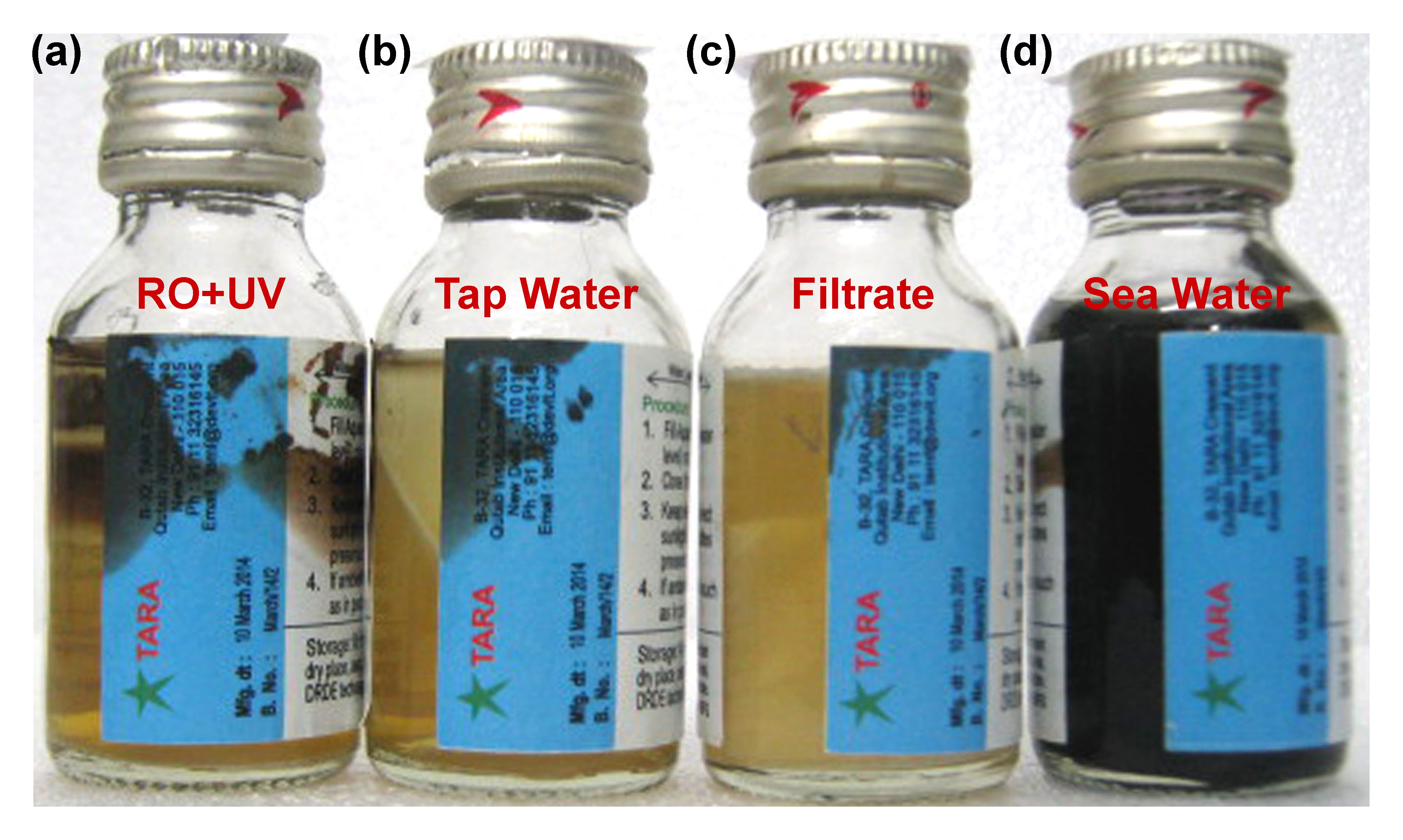


**Figure S5 (a):-** Fecal pollution check test using “Aquachek” bottle for (a) water from RO+UV commercial water purifier, (b) tap water from BMC (Brihanmumbai municipal corporation) supply, (c) filtrate from the sea water sample using filter and (d) sea water sample used as feed for (c).


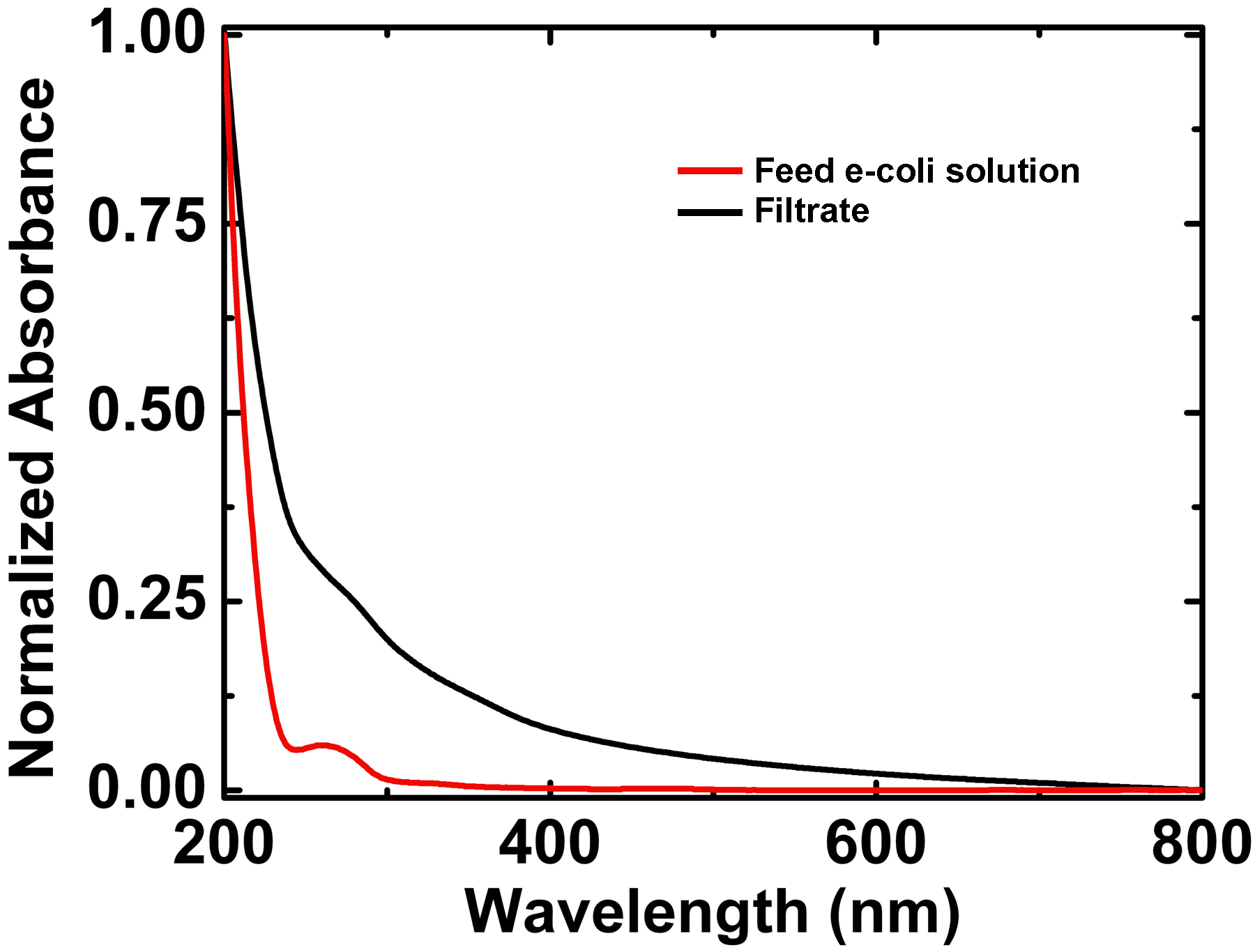


**Figure S5(b):-** (a) UV-Vis absorption spectra of the feed e-coli solution (red curve) used for filtering and the filtrate (black curve) obtained from the filter. The absence of shoulder at ~260 nm in the black curve suggests that most of the e-coli bacteria get filtered by the filter.
